# Supplementary material for: Phase angle in bioelectrical impedance analysis for assessing congestion in acute heart failure
Source: PLoS One. 2025 Jan 24;20(1):e0317333. doi: 10.1371/journal.pone.0317333 (PMC11759352; doi:10.1371/journal.pone.0317333)
Supplement: S1 Table — EF: Ejection fraction; HF: Heart failure; HFmrEF: Heart failure with mid-range/mildly reduced ejection fraction; HFpEF: Heart failure with preserved ejection fraction; HFrEF: Heart failure with reduced ejection fraction. (DOCX) [file pone.0317333.s001.docx]

**S1 Table. Phenotypes of heart failure based on ejection fraction in the patient group.**

| **Phenotype** | **N** | **% of total patients (N=50)** |
| --- | --- | --- |
| **HFpEF (EF≥50%)** | 15 | 30 |
| **HFmrEF (40%<EF<50%)** | 15 | 30 |
| **HFrEF (EF≤40%)** | 20 | 40 |

**EF**: Ejection fraction; **HF**: Heart failure; **HFmrEF**: Heart failure with mid-range/mildly reduced ejection fraction; **HFpEF**: Heart failure with preserved ejection fraction; **HFrEF**: Heart failure with reduced ejection fraction
