# Supplementary material for: Phase angle in bioelectrical impedance analysis for assessing congestion in acute heart failure
Source: PLoS One. 2025 Jan 24;20(1):e0317333. doi: 10.1371/journal.pone.0317333 (PMC11759352; doi:10.1371/journal.pone.0317333)
Supplement: S2 Table — * Measured at 50 kilo-Hertz; ** Compared using independent t-test; HFmrEF: Heart failure with mid-range/mildly reduced ejection fraction (40%<EF<50%); HFpEF: Heart failure with preserved ejection fraction (EF≥50%); HFrEF: Heart failure with reduced ejection fraction (EF≤40%). (DOCX) [file pone.0317333.s002.docx]

**S2 Table. Comparison of segmental phase angles by different phenotypes of heart failure.**

| **Segmental phase angle (º)^*^** | **Controls** | **HFpEF** | **p-value^**^** | **HFmrEF** | **p-value^**^** | **HFrEF** | **p-value^**^** |
| --- | --- | --- | --- | --- | --- | --- | --- |
| **Whole body** | 5.68 | 3.88 | <0.001 | 4.49 | <0.001 | 4.95 | 0.024 |
| **Right arm** | 5.43 | 4.09 | <0.001 | 4.59 | 0.008 | 5.02 | 0.122 |
| **Left arm** | 5.08 | 3.99 | <0.001 | 4.42 | 0.020 | 4.94 | 0.569 |
| **Trunk** | 5.33 | 3.75 | <0.001 | 4.32 | 0.004 | 4.77 | 0.144 |
| **Right leg** | 6.08 | 3.51 | <0.001 | 4.31 | <0.001 | 4.84 | 0.006 |
| **Left leg** | 6.12 | 3.39 | <0.001 | 4.25 | <0.001 | 4.73 | 0.002 |

* Measured at 50 kilo-Hertz; ** Compared using independent *t*-test; **HFmrEF**: Heart failure with mid-range/mildly reduced ejection fraction (40%<EF<50%); **HFpEF**: Heart failure with preserved ejection fraction (EF≥50%); **HFrEF**: Heart failure with reduced ejection fraction (EF≤40%)
