# Supplementary material for: Phase angle in bioelectrical impedance analysis for assessing congestion in acute heart failure
Source: PLoS One. 2025 Jan 24;20(1):e0317333. doi: 10.1371/journal.pone.0317333 (PMC11759352; doi:10.1371/journal.pone.0317333)
Supplement: S3 Table — * Partial correlation coefficient adjusted for age, sex, body mass index, and serum creatinine; BNP: log-transformed serum NT-proBNP; EI: Edema index; kHz: kilohertz; LFC: chest CT-measured lung fluid content. (DOCX) [file pone.0317333.s003.docx]

**S3 Table. Correlation between bioelectrical impedance analysis-measured phase angle and edema index and other established markers of heart failure including serum NT-proBNP and chest CT-measured lung fluid content.**

| **Coefficient ^*^**  **(p-value)** | **5 kHz** | | | **50 kHz** | | | **250 kHz** | | |
| --- | --- | --- | --- | --- | --- | --- | --- | --- | --- |
|  | **EI** | **BNP** | **LFC** | **EI** | **BNP** | **LFC** | **EI** | **BNP** | **LFC** |
| **Whole body** |  |  |  | -0.923 | -0.474 | -0.506 |  |  |  |
|  |  |  |  | (<0.001) | (<0.001) | (<0.001) |  |  |  |
| **Right arm** | -0.733 | -0.339 | -0.360 | -0.811 | -0.383 | -0.464 | -0.515 | -0.211 | -0.360 |
|  | (<0.001) | (0.005) | (0.002) | (<0.001) | (0.002) | (<0.001) | (<0.001) | (0.089) | (0.002) |
| **Left arm** | -0.717 | -0.217 | -0.316 | -0.780 | -0.271 | -0.406 | -0.406 | 0.006 | -0.264 |
|  | (<0.001) | (0.08) | (0.008) | (<0.001) | (0.028) | (0.001) | (0.001) | (0.965) | (0.027) |
| **Trunk** | -0.348 | -0.172 | -0.216 | -0.804 | -0.421 | -0.471 | -0.630 | -0.248 | -0.292 |
|  | (0.004) | (0.169) | (0.073) | (<0.001) | (<0.001) | (<0.001) | (<0.001) | (0.044) | (0.014) |
| **Right leg** | -0.895 | -0.425 | -0.410 | -0.968 | -0.537 | -0.515 | -0.851 | -0.424 | -0.394 |
|  | (<0.001) | (<0.001) | (<0.001) | (<0.001) | (<0.001) | (<0.001) | (<0.001) | (<0.001) | (0.001) |
| **Left leg** | -0.860 | -0.398 | -0.364 | -0.960 | -0.557 | -0.511 | -0.850 | -0.567 | -0.484 |
|  | (<0.001) | (0.001) | (0.002) | (<0.001) | (<0.001) | (<0.001) | (<0.001) | (<0.001) | (<0.001) |

***** Partial correlation coefficient adjusted for age, sex, body mass index, and serum creatinine; **BNP**: log-transformed serum NT-proBNP; **EI**: Edema index; **kHz**: kilo-Hertz; **LFC**: chest-CT measured lung fluid content
