# Supplementary material for: Phase angle in bioelectrical impedance analysis for assessing congestion in acute heart failure
Source: PLoS One. 2025 Jan 24;20(1):e0317333. doi: 10.1371/journal.pone.0317333 (PMC11759352; doi:10.1371/journal.pone.0317333)
Supplement: Attachment — Submitted filename: Response to Reviewers.docx [file pone.0317333.s006.docx]

Dear Academic Editor and Reviewers,

We sincerely appreciate your considerate opinions. After thorough discussions regarding the critiques, we have made revisions on our paper to reflect all of your valuable recommendations. Our point-by-point responses to the comments with revised manuscript body in blue are described as follows:

**<Response for Academic Editor>**

**-abstract should include statistical results;**

**-keywords should not contain the same words already present in the title;**

**-a hypothesis should be added in the end of introduction (it should be supported with references);**

We have updated the abstract to include statistical results and revised the keywords to avoid duplicating words from the title, as per your requirements. Additionally, we have moved the study purpose to a separate paragraph at the end of the Introduction section for improved clarity.

**-a sample size calculation or statistical power should be added in methods;**

Thank you for your comment and suggestions. As noted in our response to Reviewer 1, we have addressed the lack of a formal sample size calculation in detail. While we acknowledge that a proper sample size calculation could have strengthened the study, it was challenging to determine reference values needed for such calculations, given the exploratory nature (pilot study) of our research. We understand that it is a common limitation in early-stage, conceptual studies.

**-just like reviewers 1 mentioned, authors are welcome to use STROBE observational studies reporting guidelines https://www.strobe-statement.org/checklists/ to verify if there is an additional aspect to include or modify. For instance, described the setting and location of subject recruitment and sample size calculations;**

**-information about the procedures adopted before the body compositon analysis should be described as well as the timming of the assessment;**

**-validity of the device used can be added as well;**

In response to your comments and suggestions, we have thoroughly revised our manuscript, particularly in the Methods section, to ensure compliance with the STROBE guidelines. Additionally, we have provided detailed information regarding the procedures adopted before body composition analysis, the timing of the assessments, and the validity of the device used. The STROBE checklist has been uploaded with this revision.

**-for better clarity, authors are welcome to introduce a subsection in discussion with limitations, practical implications and directions for future studies. A final section of conclusions would also bring clarity for the reader. This topics are not mandatory in this journal, but they can improve clarity and quality of the work.**

In response to your suggestions, we have introduced a subsection dedicated to study limitations and have separated the conclusion for better clarity. However, we opted to keep the clinical implications and directions for future studies within their respective contexts to maintain logical flow throughout the manuscript.

In addition to your overall comments, we have put our best effort to meet the journal requirements as well as style requirements. Relevant updates for disclosures are included in the cover letter per the journal requirement.

Thank you again for your thoughtful comments and suggestions.

**<Response for Reviewer #1>**

**1. I suggest authors use STROBE observational studies reporting guidelines https://www.strobe-statement.org/checklists/ to verify if there is an additional aspect to include or modify. For instance, described the setting and location of subject recruitment and sample size calculations.**

As your comment, we have thoroughly reviewed our entire manuscript and revised it in greater detail to comply with the STROBE guideline. In response to your suggestions, we have uploaded the STROBE checklist with this revision.

We agree that performing a proper sample size calculation could have strengthened our study. However, as our study is exploratory in nature, closer to a pilot or preliminary investigation rather than confirmatory research, it was challenging to postulate reference values needed for sample size calculations. This difficulty is often seen in early-stage, conceptual studies (Amir *et al*., 2016; Lee *et al*., 2024; Leungratanamart *et al*., 2024). We added this issue to the study limitations in the Discussion section as follows:

(Page 15/Lines 275-281) As this study serves as a preliminary exploration of the potential utility of relatively novel markers, such as phase angle, where supporting evidence remains limited, determining an a priori sample size was not feasible, a common challenge in early-stage research [6,9,32]. To mitigate this limitation, we aimed to enroll as many participants as possible within the study period. However, expanding the study’s scope presented additional challenges due to the need for control participants to undergo chest CT scans, which expose them to unnecessary radiation, despite the low actual risk involved.

**2. This study had two objectives, but the authors presented them as one. To evaluate a screening tool, it is necessary to perform a different methodology (diagnostic test and longitudinal) and study populations with follow-up to develop acute heart failure.**

Thank you for your valuable comment. We have carefully considered this critical comment and agree that our original submission could indeed be misleading. Our intention in using terms such as ‘screening’ or ‘predicting’ was to highlight the potential role of phase angle in identifying patients at risk of acute HF, rather than implying its use for confirmatory diagnosis. In accordance to your feedback and to avoid any misleading to readers, we decided to change and clarify the title of the study as follows:

(Page 1/Lines 1-2) Phase angle in bioelectrical impedance analysis for assessing congestion in acute heart failure

Additionally, we have narrowed the focus of the study’s purpose to emphasize identifying the correlation between phase angle and markers of congestion, while explaining the clinical implications related to acute HF as follows:

(Page 5/Lines 87-79) Therefore, the purpose of this study was to evaluate the correlation between the PhA measured by BIA and previously known measures of HF, and to assess the additive value of PhA alongside the EI as an indicator for detecting acute HF.

Furthermore, we have made extensive revisions throughout the manuscript, particularly in the Discussion section, to remove any ambiguous terms and better clarify our intention. For example:

(Page 13/Lines 232-233) This suggests the potential utility of PhA as an assessment tool for congestion that indicates HF aggravation among other acute conditions.

(Page 14/Lines 258-261) However, using PhA to assess congestion is expected to facilitate frequent monitoring, enabling early detection of changes in fluid status and timely interventions, such as diuretic adjustments, which may help prevent readmissions and improve patient outcomes.

(Page 15/Lines 293-295) Its non-invasive nature and reliable accuracy underscore its potential as an additional tool for assessing fluid overload and congestion in HF patients, making it suitable for daily monitoring by nonprofessionals.

**3. The equipment used to evaluate the phase angle (InBody S10 analyzer) provides segmental measurements that are calculated or modelled to obtain total body parameters, which may not be appropriate biophysically; therefore, data from this device could be unreliable or underestimated.**

Thank you for raising this important point. We were also aware of the concerns surrounding different BIA measuring techniques, especially the use of complex regressions or modeling in some earlier BIA devices. Many of these devices assumed of a single-cylinder model, providing a single whole-body measurement, which could introduce inaccuracies.

However, the InBody S10 in our study employs a more advanced technique known as direct segmental multi-frequency bioelectrical impedance analysis (DSM-BIA). DSM-BIA allows for direct measurement of individual body segments (arms, legs, and trunk) in addition to the whole body, minimizing the potential for errors arising from generalized assumptions or modeling processes. By avoiding the error-prone calculations used in earlier techniques, DSM-BIA is known to provide more accurate and reliable data across different body segments, reducing the likelihood of misrepresenting parameters.

Furthermore, the validity and reproducibility of DSM-BIA devices, including the InBody S10, have been extensively studied. Multiple studies have demonstrated their reliability in both validation and clinical applications, with reasonable agreement across various devices from different manufacturers (Ling *et al*., 2011; Bosy-Westphal *et al*., 2017; Yang *et al*., 2017). Considering this context, we would like to say that the selection of certain device that has already been widely accepted is less likely to compromise the integrity of our findings.

In response to your concern, however, we have revised the Methods section to include additional details on the measurement procedure and device selection, as follows:

(Page 6/Lines 112-116) Two BIA parameters, PhA and EI, were measured at three different frequencies (5 kHz, 50 kHz, and 250 kHz) using the InBody S10 (InBody Co., Ltd., Seoul, Korea), a direct segmental multi-frequency BIA (DSM-BIA) device. Compared to conventional BIA techniques such as the wrist-ankle method, DSM-BIA allows for direct measurements of the whole body as well as individual segments (arms, legs, and trunk) through multiple electrodes. The validity and test-retest reproducibility of this device, along with other DSM-BIA devices, have been demonstrated in previous studies [19–21].

**4. In addition, this device's measurements at the lowest and highest frequencies can interfere with other devices, such as cardiorespiratory monitoring used in heart failure patients, giving erroneous results that the evaluator does not detect.**

We regret to mention this point more in detail. As mentioned in our original manuscript under the exclusion criteria (“patients with any type of cardiac implants, limb defects, or ambulatory difficulties were excluded”), we had already taken this issue into consideration during the study design phase to avoid any potential interference from the BIA device with other monitoring devices. Additionally, we have now provided more detailed information on the procedure in the Methods section to further clarify this aspect as follows:

(Page 6/Line 120) All metallic or conductive objects and personal items were removed to avoid interference.

**5. The validity of BIA measurements when, where, and how they were performed was not informed.**

We described more in detail regarding the measurement process as follows:

(Pages 6-7/Lines 117-123) To better capture the edematous status in acute HF, BIA was performed within 3 days of admission. The test was conducted in a dedicated testing room by a single trained technician to minimize measurement bias. Before the exam, patients rested in a supine position on an insulating bed for 15 min. All metallic or conductive objects and personal items were removed to avoid interference. Eight electrodes were attached to the thumbs, third fingers, and ankles on both sides. Patients were instructed to keep their fingers, arms, and limbs abducted to avoid electrical current interference and remain still during the analysis.

Additionally, in response to your valuable feedback on the BIA measurement, we have revised the Bioelectrical Impedance Analysis section in the Methods to provide more comprehensive details. Thank you for your insightful comments.

**6. A phase angle cut off value of 5 is very high the normal range goes from 5.03 to 7 and in reported phase angle values associated with negative outcomes <4°**

Thank you for this insightful comment. While it is challenging to establish a definitive reference value or range for phase angle, we agree that the generally accepted normal range is above 5°, which overlaps with our suggested cutoff value. However, evidence indicates that normal values can vary depending on factors such as age, gender, and underlying conditions (Mattiello *et al*., 2020). Likewise, the abnormal values also differ based on whether the condition is acute or chronic, even within patients with HF from 4.7° to 5.5° (Massari *et al*., 2016).

Although the cutoff value of 5° may appear higher, the average values in our study ranged between 4.18° and 4.61°, which fall within a reasonable range compared to similar studies, supporting the validity of our results. Since our study suggests frequent self-monitoring of PhA by patients for early detection of edematous conditions, we believe that providing a broader, albeit less precise, risk range (e.g., higher PhA) may be more advantageous in a preventive setting compared to a narrower, more exact value.

We recognize that this is a clinically important issue and have added further details to the Discussion section to address it as follows:

(Page 14/Lines 253-262) Our study demonstrated that a PhA cutoff estimation of 5º provided the maximum AUC (Table 3), which was slightly higher than the average PhA of 4.18° to 4.61° observed in the patient group. Interestingly, the sensitivity of 84% at this cutoff was higher than the sensitivity values for EI and LFC at their respective cutoffs. Studies have shown varying reference ranges for PhA, even in healthy populations [29], due to differences in age and sex, making it more challenging to establish a definitive PhA cutoff for disease conditions [18]. However, the expected implication of using PhA for assessing congestion is to enable frequent monitoring, allowing for the early detection of fluid status changes and the initiation of prompt interventions, such as adjusting diuretics, which can prevent readmissions and improve prognosis [4]. In this context, a broader risk range (*i.e.*, higher PhA), though less precise, may offer better preventive benefits than a narrower value, which might overlook patients at borderline risk.

**7. Because phase angle (PhA), edema index, and chest CT-measured lung fluid content are correlated, should not be evaluated as “predictors” because of multicollinearity.**

We agree that the measures reviewed in our study, such as PhA, EI, and chest CT-measured lung fluid content, are correlated, and indeed, the purpose of our study was to examine this correlation. We also acknowledge that correlated variables should be handled carefully, as you mentioned. However, to the best of our understanding, multicollinearity is primarily a concern when estimating coefficients through regression models, which differs from the objective of our study.

In our analysis, we used PhA and EI as variables in the ROC analysis, which aimed to assess their ability to classify study subjects according to the appropriate disease status. We did not perform point estimation of these variables' effect sizes, where multicollinearity would be more relevant.

That being said, we do agree that our original use of the term ‘predictors’ may have been misleading to some readers. Therefore, we have revised our terminology, replacing ‘predictors’ with ‘parameters’ to reflect a more neutral interpretation as follows:

(Page 7/Lines 139-143) Area under the curve (AUC) from the receiver operating characteristic analysis was calculated to compare the discriminatory value of parameters. First, unadjusted crude models using PhA or EI as single parameters and a multivariable model incorporating both PhA and EI were compared to an LFC single parameter model. Next, adjusted models including control variables were compared for sensitivity analysis.

**8. The areas under the curves should be presented with confidence intervals and a comparison made using the DeLong test.**

Thank you for your comment. We have updated Table 3 to include confidence intervals for the AUC values, as recommended.

Additionally, the DeLong test was performed for AUC comparisons in our original analysis, but we regret that this was not detailed sufficiently. We have now corrected this in the Methods section and added further details in the footnotes of the relevant figures as follows:

(Page 7/Line 143) DeLong test was used for these AUC comparisons.

(Page 22/Lines 423-424) p-values were calculated using the DeLong test for comparison of each measure versus the LFC as reference

Thank you again for your thoughtful comments.

**<Response for Reviewer #2>**

**1. It remains unclear what phenotype(s) of HF was /were included in the study.**

Thank you for your important comment. Following your advice, we reviewed our data and updated our results. Among the 50 patients with HF, there were 15 patients with preserved EF, 15 with mid-range/mildly reduced EF, and 20 with reduced EF.

Additionally, we examined whether the PhA differences observed in the HF group were consistent across these HF phenotypes. Consistent with our original findings, all subgroups demonstrated lower PhA across all segments compared to controls. Especially, whole-body and lower limb PhA were significantly lower across all phenotypes.

However, as noted in your comment #3 and our response, we were cautious about interpreting the phenotype-specific differences in PhA more in detail due to the small sample size. Therefore, we have added these findings to the Supporting information (S1 Table, S2 Table), and briefly described in Results section as follows:

(Page 8/Lines 153-155) Among the HF group, 15 patients (30%) had preserved ejection fraction (EF), 15 patients (30%) had mid-range/mildly reduced EF, and 20 patients (40%) had reduced EF (S1 Table).

(Page 10/Lines 171-172) Additionally, differences in whole-body and lower limb PhA measured at 50 kHz were robust across all HF phenotypes (S2 Table).

**S1 Table. Phenotypes of heart failure based on ejection fraction in the patient group.**

| **Phenotype** | **N** | **% of total patients (N=50)** |
| --- | --- | --- |
| **HFpEF (EF≥50%)** | 15 | 30 |
| **HFmrEF (40%<EF<50%)** | 15 | 30 |
| **HFrEF (EF≤40%)** | 20 | 40 |

**EF**: Ejection fraction; **HF**: Heart failure; **HFmrEF**: Heart failure with mid-range/mildly reduced ejection fraction; **HFpEF**: Heart failure with preserved ejection fraction; **HFrEF**: Heart failure with reduced ejection fraction

**S2 Table. Comparison of segmental phase angles by different phenotypes of heart failure.**

| **Segmental phase angle (º)^*^** | **Controls** | **HFpEF** | **p-value^**^** | **HFmrEF** | **p-value^**^** | **HFrEF** | **p-value^**^** |
| --- | --- | --- | --- | --- | --- | --- | --- |
| **Whole body** | 5.68 | 3.88 | <0.001 | 4.49 | <0.001 | 4.95 | 0.024 |
| **Right arm** | 5.43 | 4.09 | <0.001 | 4.59 | 0.008 | 5.02 | 0.122 |
| **Left arm** | 5.08 | 3.99 | <0.001 | 4.42 | 0.020 | 4.94 | 0.569 |
| **Trunk** | 5.33 | 3.75 | <0.001 | 4.32 | 0.004 | 4.77 | 0.144 |
| **Right leg** | 6.08 | 3.51 | <0.001 | 4.31 | <0.001 | 4.84 | 0.006 |
| **Left leg** | 6.12 | 3.39 | <0.001 | 4.25 | <0.001 | 4.73 | 0.002 |

* Measured at 50 kilo-Hertz; ** Compared using independent *t*-test; **HFmrEF**: Heart failure with mid-range/mildly reduced ejection fraction (40%<EF<50%); **HFpEF**: Heart failure with preserved ejection fraction (EF≥50%); **HFrEF**: Heart failure with reduced ejection fraction (EF≤40%)

**2. How did the serum NT-proBNP concentration change in observation period and did the authors evaluate the link between the phase angle and NT-proBNP.**

Yes, we evaluated the correlation between PhA and NT-proBNP, which showed a moderate correlation, particularly at 50 kHz. The results are presented in Figure 1 and supporting information S3 Table. However, as our study only measured markers at the time of admission, we were unable to collect longitudinal data to assess changes over time. We agree that tracking these changes could have provided a deeper understanding of PhA and its clinical implications. We have added this as a limitation in the study, as follows:

(Page 15/Lines 285-289) Third, the cross-sectional design of our study limited our ability to evaluate whether PhA or other markers, such as NT-proBNP, measured at a single time point or tracked over time, could offer insights into clinical outcomes and prognosis in HF patients. Further studies utilizing time-series data are needed to understand the clinical implications of PhA, both in the preclinical stage and over the course of long-term management.

**3. The patients' cohort was heterogenic and composes of AF individuals and diabetes mellitus. Did the authors investigate whether the phase angle is able to predict HF exacerbation in individuals as with lower NT-proBNP (i.e., patients with diabetes) as well as individuals with higher than expected (AF patients)?**

Thank you for raising this important issue. As you mentioned, external factors such as diabetes (DM) and atrial fibrillation (AF) can influence the expected changes in NT-proBNP levels in patients with acute HF due to their unique physiological characteristics, such as metabolic changes or atrial strain (Brunner-La Rocca *et al*., 2019). We investigated whether these phenomena were observed in our dataset, and the results are summarized in Response Table 1 and Response Figure 1:

Although not statistically significant, we found that average NT-proBNP levels were higher in patients with DM and in patients without AF, which is contrary to the expected trend based on these conditions (Response Table 1).

**Response Table 1**. Serum NT-proBNP levels by underlying conditions that are known to alter expected changes of NT-proBNP levels

|  | **N** | **Mean** | **(SD)** | **p-value** | **IQR-25%** | **Median** | **IQR-75%** |
| --- | --- | --- | --- | --- | --- | --- | --- |
| **HF patients** |  |  |  |  |  |  |  |
| with DM | 26 | 5489.4 | 7398.5 | 0.491 | 1431 | 3183.5 | 6616 |
| without DM | 24 | 4277.4 | 4766.0 | ref. | 1823.5 | 2962.5 | 5296 |
| with AF | 22 | 4745.6 | 7042.2 | 0.872 | 2143 | 3141 | 4830 |
| without AF | 28 | 5035.0 | 5665.8 | ref. | 1495.5 | 2784 | 6690.5 |
| **Controls** |  |  |  |  |  |  |  |
| with DM | 6 | 97.2 | 26.2 | 0.151 | 100 | 106 | 112 |
| without DM | 14 | 64.1 | 50.8 | ref. | 27.88 | 51.99 | 74.54 |
| with AF | 0 | - | - | NA | - | - | - |
| without AF | 20 | 74.0 | 46.8 |  | 43.88 | 60.17 | 106 |

Additionally, the correlation between PhA and NT-proBNP remained similar to that of the original cohort in patients with DM, but this correlation lost significance in patients with AF (Response Figure 1).

**(a)** **(b)**

**Response Figure 1**. Partial correlation between the phase angle and serum NT-proBNP level in patients (a) with diabetes and (b) with atrial fibrillation.

We agree that your point holds clinical significance; however, due to the small sample size and the limitations of our data, we are hesitant to draw additional conclusions or perform subgroup analysis. So, we decided not to extend this analysis further in our manuscript to avoid overinterpretation at this point.

Thank you again for your thoughtful comments.
