# Supplementary material for: Phase angle in bioelectrical impedance analysis for assessing congestion in acute heart failure
Source: PLoS One. 2025 Jan 24;20(1):e0317333. doi: 10.1371/journal.pone.0317333 (PMC11759352; doi:10.1371/journal.pone.0317333)
Supplement: Attachment — Submitted filename: Response to Academic Editor.docx [file pone.0317333.s007.docx]

Dear Academic Editor,

We sincerely appreciate your considerate opinions. Our point-by-point responses to the comments with revised manuscript body in blue are described as follows:

**Journal Requirements:**

**Please review your reference list to ensure that it is complete and correct. If you have cited papers that have been retracted, please include the rationale for doing so in the manuscript text, or remove these references and replace them with relevant current references.**

→ We have carefully reviewed the reference list for completeness and retraction status. At this time, no retractions are noted, and the original reference list remains unchanged. Minor changes such as title change have been updated.

**Additional Editor Comments:**

**- please revise your writing and avoid doing it in the first person;**

→ Following the necessary changes in this round of minor revisions, we also went through language editing service for a secondary review to improve the flow and readability of the manuscript, as well as to ensure compliance with the request to avoid first-person tense. The certificate of language editing is attached in the revised file. We appreciate the opportunity to further enhance the quality of our submission.

**- a hypothesis should be added in the end of introduction (it should be supported with references);**

→ We have added the study hypothesis in the end of introduction as follows:

(Page 5/Lines 89-91) It was hypothesized that the phase angle is significantly correlated with NT-proBNP and LFC and may serve as a useful parameter for assessing congestion in patients with acute heart failure.

**- information about the procedures adopted before the body compositon analysis should be described as well as the timming of the day for the assessment;**

→ We regret not providing more detail on this point earlier. In fact, no additional procedures were adopted before the BIA testing, aside from resting on the testing bed, which has already been mentioned in the manuscript. To clarify this further, we have added some more details to the corresponding section, as follows:

(Page 7/Lines 118-119) BIA was performed within 3 days of admission, and no specific preparatory procedures were required.

**- as suggested, authors already introduced a subsection in discussion with limitations title, but this title should also include practical implications and directions for future studies.**

→ Thank you for your valuable comment and suggestion. As explained in our previous response letter, we opted to keep the clinical implications and directions for future studies within their respective contexts to maintain logical flow and readability throughout the discussion section (For example, *“This suggests the potential utility of PhA as an assessment tool~”* in lines 235-236, or *“Further studies utilizing time-series data are required~”* in lines 293-294).

However, to address your suggestion and ensure compliance as well, we have introduced the subtitle "Limitations" at the end of the discussion section, where the study limitations were originally structured separately.

We kindly ask for your understanding as we have put our best effort to balance these adjustments with the coauthors’ intentions.

Again, we deeply appreciate for your thoughtful comments.
