# Supplementary material for: Phase angle in bioelectrical impedance analysis for assessing congestion in acute heart failure
Source: PLoS One. 2025 Jan 24;20(1):e0317333. doi: 10.1371/journal.pone.0317333 (PMC11759352; doi:10.1371/journal.pone.0317333)
Supplement: Attachment — Submitted filename: Response to Academic Editor.docx [file pone.0317333.s008.docx]

Dear Academic Editor,

We sincerely appreciate your considerate opinions. Our point-by-point responses to the comments with revised manuscript body in blue are described as follows:

**Additional Editor Comments:**

**The majority of the comments were addressed with the exception of the following:**

**- information about the procedures adopted before the body compositon analysis should be described as well as the timming of the day for the assessment;**

**→ We regret not providing more detail on this point earlier. In fact, no additional procedures were adopted before the BIA testing, aside from resting on the testing bed, which has already been mentioned in the manuscript. To clarify this further, we have added some more details to the corresponding section, as follows:**

**(Page 7/Lines 118-119) BIA was performed within 3 days of admission, and no specific preparatory procedures were
required.**

**I'm not sure about your answer. As the authors should know, the body composition analysis, using InBody S10, includes several cautions before measurement. The authors stated that no specific preparatory procedures were required. However, such statement makes me wondering if the authors have enough expertise on using this device or measuring body compositon in general. So, I will provide another chance to clarify this (Cautions Before Measurement) as well as the timming of the day for assessment before moving with a final decision on your work. Authors must provide all information so other researchers can replicate your study in the exact same way.;**

→ Thank you for your valuable feedback. We regret any inconvenience caused by not resolving this issue earlier. It seems there may have been a difference in understanding regarding the *procedures adopted before the testing*.

We initially understood your mention of "procedures" as referring to *medical* interventions (*e.g.*, administering pre-operative antibiotics before surgery). For this reason, we previously replied that no preparatory *medical* procedures were required for BIA testing. And *non-medical* precautions necessary before the test were not included in our previous description of the BIA measurement steps, because we assumed these would be already familiar to physicians using the BIA in their practice.

However, we strongly agree that the manuscript should provide sufficient detail to allow any researchers to replicate our approach. Therefore, after carefully discussing your comment and reviewing other studies that incorporated BIA testing, we have thoroughly revised the BIA measurement section as follows:

(Page 7/Lines 118-126) Bioelectrical impedance analysis (BIA) was conducted within 3 days of admission to assess the edematous status of patients with acute HF. To reduce measurement bias, all tests were performed in a dedicated testing room by a single trained technician. Standardized pre-test precautions were implemented starting the day prior to the procedure. Patients were instructed to abstain from exercise, bathing, or activities leading to excessive sweating for 6–12 hours before the test and to avoid alcohol or caffeine for 24 hours prior. In the morning of the test, patients were asked to fast, empty their bladder, and remove all metallic or conductive items to prevent interference. BIA was performed with patients lying in the supine position on an insulated bed for 15 minutes to minimize measurement variability caused by fluid shifts.

We have put a great deal of thought and effort into understanding and addressing your comments as thoroughly as possible. However, we still have some concerns that our revisions may not fully reflect the intent of your feedback.

If you feel that our revisions have not adequately captured your intentions, we kindly ask for your understanding and would greatly appreciate it if you could provide more specific examples to guide us, allowing us another opportunity to make further improvements.

Again, we deeply appreciate for your thoughtful comments.
